# Cost effectiveness of vildagliptin versus glimepiride as add-on treatment to metformin for the treatment of diabetes mellitus type 2 patients in Greece

**DOI:** 10.1186/s12962-017-0082-7

**Published:** 2017-09-06

**Authors:** Hara Kousoulakou, Magdalini Hatzikou, Varvara Baroutsou, John Yfantopoulos

**Affiliations:** 1 University of Peloponnese, Damaskinou & Kolokotroni, 20100 Corinth, Greece; 2 Novartis Hellas SACI, National Rd, No 1, 12th km, Metamorphosis, 14451 Athens, Greece; 3 National and Kapodistrian University of Athens, 45 Akadimias, 10672 Athens, Greece; 4 School of Economics and Political Science , Athens, Greece

**Keywords:** Vildagliptin, Type 2 diabetes mellitus, Cost-effectiveness, Greece

## Abstract

**Objectives:**

This study was designed to assess the cost-effectiveness of vildagliptin versus glimepiride as add-on to metformin in the management of type 2 diabetes mellitus (T2DM) patients in the Greek healthcare setting.

**Methods:**

A cost-effectiveness model was designed, using MS Excel, to compare two treatment strategies. Strategy 1 consisted of first-line metformin, followed by metformin + vildagliptin in second-line, while strategy 2 consisted of first line metformin, followed by metformin + glimepiride in second line. Subsequent lines were the same in both strategies and consisted of metformin + basal insulin and metformin + basal + rapid insulin. Clinical data and utility decrements relating to diabetes complications were taken from the published literature. Only direct medical costs were included in the analysis (cost base year 2014), and consisted of drug, adverse events and comorbidity costs (taken from local officially published sources and the literature). The perspective adopted was that of the Social Insurance Fund. The time horizon was lifetime, and future costs and outcomes were discounted at 3.5% per annum.

**Results:**

Adding vildagliptin to metformin increased drug costs compared with adding glimepiride to metformin (€2853 vs. €2427, respectively). However, this increase was offset by a decrease in the costs of associated comorbidities (€4393 vs. €4539) and adverse events (€2757 vs. €3111), resulting in a lower total cost of €74 in strategy 1 compared with strategy 2. Comorbidities were the largest cost component in both strategies, accounting for 43.9 and 45.0% in strategies 1 and 2, respectively. Strategy 1 was also associated with increased life-years (LYs, 0.11) and quality-adjusted life-years (QALYs, 0.11) compared with strategy 2. Strategy 1 is therefore dominant, as it is associated with both lower overall costs and increased effectiveness.

**Conclusions:**

Vildagliptin as add-on treatment to metformin in the management of T2DM in Greece appears to be dominant versus. glimepiride in terms of both cost per LY and cost per QALY gained.

## Background

Type 2 diabetes mellitus (T2DM) comprises 90% of people with diabetes around the world, and is largely the result of excess body weight and physical inactivity [1]. Over the past decades, T2DM has become an epidemic [2] and affects about 6% of the adult population in the western world [3]. In Greece, the projected prevalence of T2DM in 2002 was estimated at 7.6% in men and 5.9% in women [4].

T2DM is a costly disease. It is associated with significant burden due to specific diabetes-related microvascular complications, increased risk of macrovascular complications (ischemic heart disease, stroke and peripheral vascular disease), blindness, renal failure and amputations [5–7]. The American Diabetes Association (ADA) estimated the total cost of diabetes in the US at $174 billion in 2007 [8]. A recent Greek study estimated the total annual cost per patient for managing T2DM in Greece at €7111 [9].

International guidelines recommend therapy in individuals with T2DM to target HbA1c ≤7% to minimize micro- and macrovascular risk [10]. A target of HbA1c ≤6.5% may be considered in some patients with T2DM, but this must be balanced against the risk of hypoglycemic events [11, 12], which represent a significant economic burden [13].

In a randomized, open-label, comparative study vildagliptin–metformin treatment provided blood glucose control efficacy comparable to that of glimepiride–metformin treatment and resulted in better adverse event profile with lower risks of hypoglycemia and weight gain [14]. Another study showed that when metformin monotherapy fails to maintain sufficient glycaemic control, the addition of vildagliptin provides comparable efficacy to that of glimepiride, with a favourable safety profile and significant reduction in hypoglycaemia compared with glimepiride [15].

Therefore, it was deemed necessary to investigate the additional costs associated with the additional benefits of vildagliptin–metformin in the management of T2DM, since payers are increasingly interested in the economic evaluation of new health technologies. This study aimed at assessing the cost effectiveness of vildagliptin versus glimepiride as add-on to metformin in the Greek healthcare setting.

## Methods

### Model design

A cost-effectiveness patient simulation model was developed in Excel^®^ based on the risk equations from the UK Prospective Diabetes Study (UKPDS) Outcomes model [16]. During each cycle a patient could experience any of the following complications: ischemic heart disease, myocardial infarction, congestive heart failure, renal failure, stroke, amputation. The model is described in detail elsewhere [17].

A cohort of 10,000 patients who failed to achieve glycemic control with metformin monotherapy entered the model. Baseline characteristics were assumed to be the same for both cohort models and were based on the Diabetes Atlas of the International Diabetes Federation [18]. Mean patient age at model start was 63 years.

Two treatment strategies were compared in the model: strategy 1 in which vildagliptin is added to metformin in 2nd line treatment and strategy 2 in which glimepiride is added to metformin in 2nd line treatment (Table 1). The HbA1c threshold (7%) based on which patients were switched to the next in line treatment was taken from the Hellenic Diabetes Association guidelines [19].

**Table 1:** Treatment strategies

	Strategy 1	Strategy 2
1st line treatment	Metformin	Metformin
2nd line treatment	Metformin + vildagliptin	Metformin + glimepiride
3rd line treatment	Metformin + basal insulin	Metformin + basal insulin
4th line treatment	Metformin + intensive insulin	Metformin + intensive insulin

The model’s time horizon was that of a lifetime and outcomes assessment criteria were Quality Adjusted Life Years (QALYs) and Life Years (LYs). Future costs and outcomes were discounted at 3.5% per annum, since cost-effectiveness results should reflect the present value of the stream of costs and benefits accruing over the time horizon of the analysis [20].

### Model inputs

#### Clinical data

Clinical data on drugs’ safety and efficacy, and in particular HbA1c reduction, changes in weight, and incidence rate of hypoglycemic events were extracted from a head-to-head clinical trial of vildagliptin and glimepiride in combination with metformin and a published meta-analysis of long-acting insulin analogues versus NPH human insulin [15, 21] (Table 2). Outcomes data were extrapolated beyond the trial period with the use of the UKPDS model [16].

**Table 2:** Efficacy and safety profiles of modelled interventions

	Metformin^a^	Meformin + glimepiride^a^	Metformin + vildagliptin^a^	Metformin + basal insulin^b^	Metformin + intensive insulin^b^
HbA1c initial absolute 1 year change	−1.03	−0.53	−0.44	−1.10	−1.10
Symptomatic Hypoglycaemia Risk (per year), %	0.00	16.20	1.66	16.20	16.20
Severe Hypoglycaemia Risk (per year), %	0.00	1.38	0.00	3.30	3.30
Weight gain (kg/year)	0.00	1.56	-0.23	1.70	1.70

^a^Source: Ferrannini et al. [15]

^b^Source: Monami et al. [21]

#### Cost data

The model only considered direct medical costs, which consisted of pharmaceutical, adverse event and comorbidity costs. The perspective adopted was that of the Social Insurance Funds (SIFs) and the cost base year was 2014.

Drug unit costs were based on the Positive Reimbursement List [22]. Annual costs were estimated based on daily dosages and frequency of administration included in the drugs’ Summary of Product Characteristics (SPCs) (Table 3).

**Table 3:** Drug costs

	Annual cost (€)
Metformin	42.10
Metformin + vildagliptin	593.86
Glimepiride	35.83
Metformin + glimepiride	77.93
Basal insulin	539.16
Metformin + basal insulin	581.26
Intensive insulin	494.76
Metformin + intensive insulin^a^	1118.11

^a^The cost of metformin + intensive insulin is calculated on top of the cost of metformin + basal insulin

The cost of monitoring insulin was based on the maximum number of glucose testing strips per patient per month and the respective prices reimbursed by the National Organization for Health Care Services Provision (EOPYY) [23, 24]. The annual cost for patients receiving insulin (basal or intensive) was estimated at €720, while the respective cost for patients on anti-diabetic treatment without receiving insulin was estimated at €180 (Table 4).

**Table 4:** Insulin monitoring costs per year

	Monitoring cost (€)
Metformin	180
Meformin + glimepiride	
Metformin + vildagliptin	
Metformin + basal insulin	720
Metformin + intensive insulin	

The unit costs associated with the management of diabetic complications during first year of treatment, including the cost of severe and non-severe hypoglycemia, were obtained from the Greek Diagnosis Related Groups (DRGs) tariffs [25] and the study by Maniadakis and colleagues [26] (Table 5).

**Table 5:** First year and follow up costs

	First year event cost (€)	Follow up costs after first year (€)	Source
Ischaemic heart disease	1118	933	DRG code: N29M & calculations based on data from Maniadakis et al. [26]
Myocardial infarction (non fatal)	2724	1542	DRG code: K10X & calculations based on data from Maniadakis et al. [26]
Myocardial infarction (fatal)	3924	–	DRG code: K10X + cost of death €1200 [26]
Congestive heart failure	1868	279	DRG code: K42M & calculations based on data from Maniadakis et al. 2005 [26]
Stroke (non fatal)	2625	2100	DRG code: N30Mb & calculations based on data from Maniadakis et al. [26]
Stroke (fatal)	2855	–	DRG codes: N30A + N30Ma
Amputation	3608		DRG code: K13M
Hypoglycemia (non-severe)	50		Based on clinical expert input (approx. the cost of a physician consultation)
Hypoglycemia (severe)	1735		DRG code: Θ20Μ
Renal failure	36,026	21,721	Stafylas et al. [28]

Patient follow-up costs after first year were all calculated based on data from the study by Maniadakis et al. [26] and input from a clinical expert. The estimated costs were subsequently inflated with the Health Price Index [27] to reflect 2014 prices. The cost of renal failure (€36,026 during first year and €21,721 after first year) was obtained from the study by Stafylas et al. [28].

#### Utility data

The utility value for diabetic patients without complications (0.780), as well as the utility decrements relating to ischemic heart disease, myocardial infarction, congestive heart failure, stroke, and amputation were derived from the study by Clarke and colleagues [29]. Disutility data associated with renal failure were obtained from the study by Manns et al. [30], while respective data for weight increase were based on the Dennett et al. study [31] (Table 6).

**Table 6:** Utility decrements

Event	Values	Reference
Ischemic heart disease	−0.090	[29]
Myocardial infarction (non fatal)	−0.055	[29]
Congestive heart failure	−0.108	[29]
Stroke (non fatal)	−0.164	[29]
Amputation	−0.280	[29]
Renal failure	−0.379	[30]
Symptomatic hypoglycemia risk	−0.014	[32]
Severe hypoglycemia	−0.047	[32]
Weight increase (per kg)	−0.002	[31]

### Sensitivity analysis

In order to test the robustness of model results, univariate sensitivity analyses were conducted in key variables surrounded by uncertainty. In particular, the following variables were allowed to vary in ranges based on the literature: the HbA1c threshold (between 6.5 and 7.5% [10]), utility and disutility scores (between 95% confidence intervals [29]), and discount rates for costs and benefits (between 0 and 8% [33]).

## Results

The addition of vildagliptin to metformin (strategy 1) increased pharmaceutical cost compared with the addition of glimepiride to metformin (strategy 2) by €426 (from €2427 to €2853). However, this was offset by a decrease in the associated comorbidity and adverse event costs, resulting in a lower total cost in strategy 1 (€10,003) compared with strategy 2 (€10,077) (Table 7).

**Table 7:** Mean per patient costs

	Strategy 1	Strategy 2
Pharmaceutical cost	€2853	€2427
Comorbidities costs	€4393	€4539
Ischaemic heart disease cost	€371	€ 343
Myocardial infarction cost	€1235	€1242
Congestive heart failure cost	€340	€316
Stroke cost	€1168	€1186
Amputation cost	€36	€30
Renal failure cost	€1243	€1422
Adverse events costs	€2757	€3111
Non-severe hypoglycemia cost	€19	€29
Severe hypoglycemia cost	€126	€179
Monitoring costs	€2611	€2903
Total costs	€10,003	€10,077

**Fig. 1:**
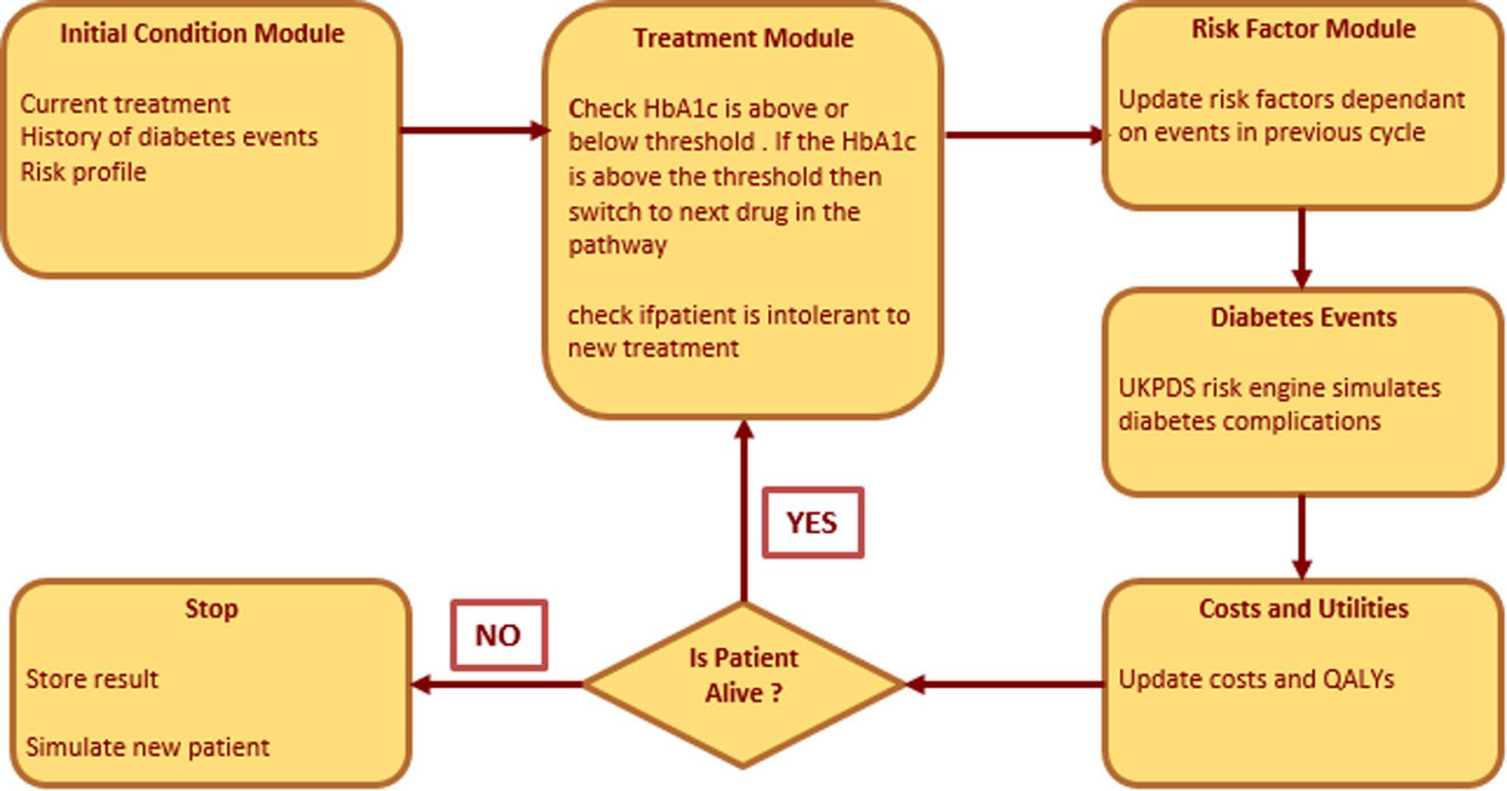
An overview of the model structure (Source: Clarke et al. [16])

Comorbidity costs represented the largest cost component in both strategies (43.9 and 45.0% in strategies 1 and 2, respectively), while drug costs accounted for 28.5 and 24.1% of total costs in strategies 1 and 2, respectively (Fig. 1).

**Fig. 2:**
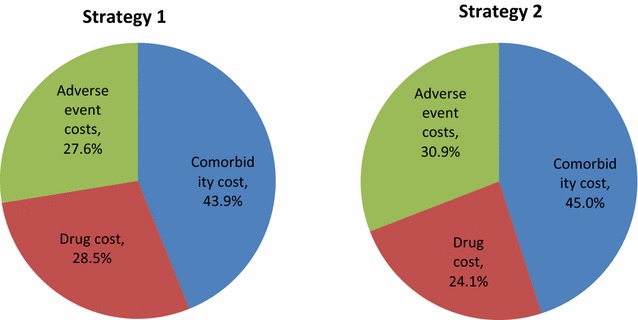
Cost breakdown

Apart from lower overall costs, strategy 1 was also associated with increased LYs (8.07 vs. 7.96 in strategy 2) and QALYs (6.30 vs. 6.19 in strategy 2) (Table 8). Therefore, strategy 1 appears to be dominant, as it is associated with both lower overall costs and increased effectiveness (Fig. 2).

**Table 8:** Basecase results

	Strategy 1	Strategy 2	Difference (strategy 1 vs. strategy 2)
Total costs	€10,003	€10,077	−€74
QALYs	6.30	6.19	0.11
Life years	8.07	7.96	0.11
ICER (cost per QALY gained)			Dominant
ICER (cost per LY gained)			Dominant

### Sensitivity analysis results

One-way sensitivity analysis confirmed dominance of vildagliptin in most variations of the parameters tested. Model results were most sensitive to changes in the HbA1c threshold. In particular, increasing the threshold from 7.0 to 7.5% resulted in vildagliptin being cost-effective rather than dominant, however with a very low incremental cost-effectiveness ratio (ICER = €1041 per QALY gained). Variations of disutility values for comorbidities did not impact significantly model results.

## Discussion

T2DM is a chronic disease, with significant economic and social implications globally. It has been estimated that 347 million people suffer from diabetes worldwide [34], and prevalence exhibits an increasing trend [35]. The annual direct cost of T2DM in eight European countries was estimated at €29 billion, with an average cost per patient of €2834 [36]. Therefore, the disease management armamentarium requires effective treatments that can control disease related expenditure. The present study assessed the costs and outcomes of adding vildagliptin to metformin in the management of T2DM in the Greek health care setting and showed that vildagliptin is associated with lower overall costs and increased health outcomes, both in terms of LYs and QALYs gained.

The study further showed that the largest cost component was the management of diabetes-related comorbidities, accounting for approx. 44% of total costs. This is confirmed by another Greek study which found that management of diabetes related comorbidities accounted for 48% of costs, while pharmaceutical treatment accounted for 35.9% [9]. The high cost of managing diabetes comorbidities is further illustrated in the international literature. In the US, mean adjusted total costs for cardiac arrest episodes, cerebrovascular disease with stroke, hypoglycemia, complication, and renal failure episodes have been estimated at $16,435; $4558; $445; $5675 and $8765, respectively [37].

The second most significant cost component was the management of adverse events. Hypoglycemia is a common T2DM adverse events and is considered the key limiting step for optimizing glycemic control [17]. The international literature has shown that both severe and non-severe hypoglycemia incur substantial healthcare costs, and failure to account for these costs may underestimate the value of management strategies that minimize hypoglycemia risk [13]. In the Greek health care setting, severe hypoglycemia costs €1735 per event and is associated with an average hospitalization length of stay of 18 days [25]. Based on the findings of the present study, adding vildagliptin to metformin is associated with reduced costs for both non-severe (by 34.5%) and severe (by 29.5%) hypoglycemia compared with adding glimepiride to metformin.

The present study had several limitations. First, patient demographics did not reflect the Greek diabetic patient population, but were based on global data. In addition, the model only direct costs were considered in the analysis, reflecting only partly the economic burden of the disease. The ADA has estimated that of the total cost associated with diabetes in the US, 66.7% is attributed to direct medical costs and 1/3 to reduced productivity [8], thus indirect costs consist a significant cost component.

In addition, the current analysis did not incorporate a number of diabetes-related comorbidities, such as peripheral neuropathy, ulceration and blindness, which are expected to impact significantly the total burden of the disease. Therefore, we can argue that the estimated costs in this study underestimate true disease burden. However, it is expected that inclusion of these comorbidities would not have impacted differently the two model arms (strategy 1 vs. strategy 2), as these would increase proportionately the management cost in both arms, and therefore, would not change model results.

The model which was used in the current economic evaluation analysis has also run with cost inputs from the Portuguese health care setting, where it showed that vildagliptin as add on to metformin is a cost-effective treatment option [17]. Adapting costs to the Greek health care system further favored vildagliptin. Such differences in the model outcomes are anticipated since both the resource use and related unit costs differ from country to country. This is confirmed by the INSTIGATE study, which estimated direct costs in five European countries and showed that the structure of direct cost varies considerably across countries, reflecting the differences in the management, resource use and prices across different health care systems [38].

Decisions in health care need to be based on local data and methods that take into consideration all cost components and not just pharmaceutical costs. The present study showed that although the addition of vildagliptin to metformin increased pharmaceutical costs, it led to a reduction in the overall costs, due to the reduction in the other cost components. Thus, the cost-effectiveness approach implemented could be considered as appropriate, since it adequately represents the complete picture of disease burden in the local setting.

## Conclusion

Vildagliptin as add-on treatment to metformin in the management of T2DM in Greece appears to be dominant versus glimepiride in terms of both cost per LY and cost per QALY gained. Decisions in health care need to be based on local data and methods that take into consideration all cost components and not just pharmaceutical costs. The present study satisfied both these criteria and produced strong results that should be taken into consideration in the decision making process by Health Authorities.

## Abbreviations

ADA: American Diabetes Association
DRGs: diagnosis related groups
EOPYY: National Organization for Health Care Services Provision, Greece
HbA1c: glycated haemoglobin
LY: life year
NPH: neutral protamine hagedorn
QALY: quality adjusted life year
SIF: social insurance fund
SPC: summary of product characteristic
T2DM: type 2 diabetes mellitus
UKPDS: UK Prospective Diabetes Study
